# Fungal Diversity in Two Wastewater Treatment Plants in North Italy

**DOI:** 10.3390/microorganisms10061096

**Published:** 2022-05-25

**Authors:** Simone Buratti, Carolina Elena Girometta, Rebecca Michela Baiguera, Barbara Barucco, Marco Bernardi, Giuseppe De Girolamo, Maura Malgaretti, Desdemona Oliva, Anna Maria Picco, Elena Savino

**Affiliations:** 1 Department of Earth and Environmental Sciences, University of Pavia, Via Sant’Epifanio 14, 27100 Pavia, Italy; simone.buratti01@universitadipavia.it (S.B.); rebeccamichela.baiguera01@universitadipavia.it (R.M.B.); annamaria.picco@unipv.it (A.M.P.); elena.savino@unipv.it (E.S.); 2 A2A Ciclo Idrico, Via Lamarmora 230, 25124 Brescia, Italy; barbara.barucco@a2a.eu (B.B.); giuseppe.degirolamo@a2a.eu (G.D.G.); maura.malgaretti@a2a.eu (M.M.); 3 CAP Holding Spa, Centro Ricerche Salazzurra, Via Circonvallazione Est, 20054 Segrate, Italy; marco.bernardi@gruppocap.it (M.B.); desdemona.oliva@gruppocap.it (D.O.)

**Keywords:** urban wastewater, fungi, diversity, depuration

## Abstract

In urban wastewater treatment plants, bacteria lead the biological component of the depuration process, but the microbial community is also rich in fungi (mainly molds, yeasts and pseudo-yeasts), whose taxonomical diversity and relative frequency depend on several factors, e.g., quality of wastewater input, climate, seasonality, and depuration stage. By joining morphological and molecular identification, we investigated the fungal diversity in two different plants for the urban wastewater treatment in the suburbs of the two major cities in Lombardia, the core of industrial and commercial activities in Italy. This study presents a comparison of the fungal diversity across the depuration stages by applying the concepts of α-, β- and ζ-diversity. Eurotiales (mainly with *Aspergillus* and *Penicillium*), Trichosporonales (*Trichosporon sensu lato*), Saccharomycetales (mainly with *Geotrichum*) and Hypocreales (mainly with *Fusarium* and *Trichoderma*) are the most represented fungal orders and genera in all the stages and both the plants. The two plants show different trends in α-, β- and ζ-diversity, despite the fact that they all share a crash during the secondary sedimentation and turnover across the depuration stages. This study provides an insight on which taxa potentially contribute to each depuration stage and/or keep viable propagules in sludges after the collection from the external environment.

## 1. Introduction

Wastewater treatment technology has a long story; the first tests on depuration by activated sludges were attempted by Ardern and Lockett in 1914 [1].

According to Italian law (Decreto Legislativo 3 Aprile 2006, n. 152) [2], currently, wastewater depuration discriminates among urban wastewater (domestic wastewater possibly mixed with industrial wastewater and rainwash water), domestic wastewater (from domestic activities and human metabolism only) and industrial wastewater (from any productive and/or commercial activity, different from domestic activity and rainwash water).

As schematized by ISPRA (Istituto Superiore per la Protezione e la Ricerca Ambientale) [3], a typical treatment plant for urban and domestic wastewater treatment is composed of different sectors basically referred to as preliminary treatment (debris and oil removal), primary treatment (reduction of total suspended solids), secondary treatment (reduction of biodegradable organic matter and colloids by activated sludge), tertiary treatment (reduction of nutrients, mainly nitrogen and phosphorous, which have not been removed yet by microbial metabolism), and disinfection (to reduce microbes before final discharge in stream/river). The plant sectors are therefore different from each other as concerns the quantity and composition of suspended solid particles, pH, microbial competition, dissolved O_2_, C/N ratio, fluid perturbation/agitation, residence time of the water and sludges [4,5,6].

Based on the above, wastewater depuration plants can host different microbial (and fungal) communities in different environmental conditions depending on the peculiar structure of the plant itself, climate, land cover and human activities in the catchment area and number of inhabitants [7,8,9,10]. The latter variable is related to the definition of “population equivalent”. A population equivalent of one person means “the organic biodegradable load having a five-day biochemical oxygen demand (BOD5) of 60 g of oxygen per day” [2,11]. The population equivalent is a basic unit to size a treatment plant and to provide it with the most suitable technology; this also affects the composition and structure of the whole microbial community.

To date, the depuration stages that significantly involve a biological activity, i.e., the activated sludges, rely on selected bacteria strains naturally mixed with autochthonous ones. Bacteria are assumed to be the most efficient and easily self-sustainable microbes that are able to crash the dissolved nitrogen in the enormous volumes of wastewater to be treated daily in urban and metropolitan areas [12,13]. On the other hand, neither fungi- nor algae-based technologies have been applied yet beyond laboratory scales despite their great potential [14].

Fungi are a well-represented component of the wastewater microbial community that can prove to be very useful and exploitable organisms thanks to their multiple capabilities [15]. Fungi can easily adapt to hostile environments and rapidly changing conditions, for instance different types of municipal and industrial wastewaters, sites strongly polluted by hydrocarbons, acid substrates, or low level of oxygen [4,16,17]. Fungi have been studied and exploited for their production of extra-cellular enzymes (e.g., laccase and peroxidase) capable of degrade complex and potentially hazardous molecules as pesticides, hydrocarbons, dyes, and pharmaceuticals [18,19,20,21,22,23]. Some species can also accumulate and bio-concentrate heavy metals and other elements [24,25,26,27].

Even if many studies are still needed, mycoflora in wastewater treatment plants could help the denitrification process, the removal of nutrients and the reduction of suspended solids. Hyphae of filamentous fungi tend to strengthen sludge flocks, making them larger and with irregular shapes and thus improving the active sludge process [28].

The fungal community in wastewater environments is highly variable, but a core of common shared genera is reported [5,29,30,31]. *Penicillium*, *Candida* and *Geotrichum* species are the most represented followed by a more variable group with *Trichoderma*, *Trichosporon* and *Rhodotorula*. Fungal taxa variation in treatment plants also depends on season and temperature: fungal diversity seems to differ between summer and winter season. Some taxa such as *Penicillium*, *Trichoderma*, *Acremonium* and *Aspergillus* are more represented in the warm months [32,33].

Taking all of this into consideration, the aim of the present work was to qualitatively characterize the fungal diversity at different stages of the depuration process in an area never investigated before. Two plants for the treatment of urban wastewater located in Lombardia, the most densely populated region and the core of productive and commercial activities in Italy, were chosen. The treatment plants in metropolitan and peri-metropolitan areas thus receive remarkable inputs throughout the whole year [34] and provide significant study cases for highly inhabited areas. Besides, the study area has a subcontinental climate with a sharp difference between summer and winter seasons that can influence fungal diversity as well.

Such a characterization aims therefore to provide a scenario of the variation in diversity patterns across the different environmental conditions in the depuration process, pointing out which taxa are the most represented or unexpected instead. This work on ecological diversity in Italian plants is preliminary to subsequent studies on the possible functional role of the fungal species present.

## 2. Materials and Methods

### 2.1. Structure of Wastewater Treatment Plants

The following treatment plants for urban wastewater were examined (the full names are not available due to security reasons):

- Plant 1, managed by CAP Holding; this plant is located in the South-West sector of the Metropolitan City of Milan; it caters for a population equivalent of 320,000 people and treats an average wastewater volume of 100,000 m^3^ day^−1^;
- Plant 2, managed by A2A Ciclo Idrico; this is located in Eastern Lombardia; it caters for a population equivalent of 296,000 people and treats an average wastewater volume of 70,000 m^3^ day^−1^.

**Figure 1:**
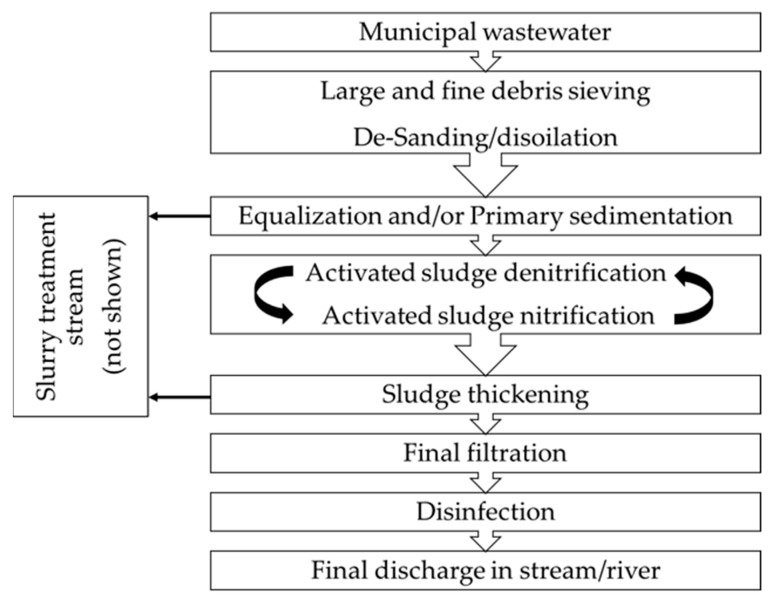
Basic scheme of water treatment in the plants. Only the water treatment stream is shown in detail.

A basic scheme of the water treatment process is reported in Figure 1.

The structures of Plant 1 and Plant 2 are slightly different from each other; therefore, the two sampling transects do not perfectly overlap and only some stages (namely, the activated sludge) are properly comparable. The two plants were consequently analyzed separately; the codes corresponding to each depuration stage in the Plants are schematized in Table 1.

### 2.2. Sampling and Isolation in Pure Culture

Samples of water and samples of sludge (i.e., water with 5–8 g L^−1^ solids suspension) were collected between November 2018 and May 2020. Samples were manually shaken for at least 1 min per bottle in order to resuspend all the particulate and homogenize the propagules distribution. Serial dilution in physiological solution (NaCl 0.9%) was axenically performed by using 1 mL as the basic unit according to the scheme in Table 2.

Bulk and diluted samples were spread in triplicate onto PDA (potato-dextrose-agar, Biokar Diagnostics), 15 cm diameter Petri dishes and incubated in the dark at room temperature for 28 days. PDA was prepared according to the manufacturer’s instructions (Biokar Diagnostics, 3.9%) and 150 ppm chloramphenicol (Fagron) were added before autoclave sterilization. Mycofloristic surveys were performed weekly to allow the propagules to overcome any latency period.

In every weekly survey, real-time approximate identification (morphotype approach) based on morphology was carried out by means of stereomicroscope (Zeiss Stemi 2000-C) and optical microscope (Zeiss Axioplan). The morphotype approach represents a first, basic step to organize the identification workload when dealing with apparently numerous taxa and little survey time, either for fungi or other organisms [35,36].

At least two cultures per morphotype were isolated in a glass tube containing PDA (as above), corked with raw cotton and incubated at room light and temperature. Pure cultures were morphologically checked to validate the morphotype.

### 2.3. Molecular Identification of Selected Strains

Based on the strain set obtained as above, at least one isolated morphotype per each plant was selected for further molecular identification.

DNA extraction was obtained by means of a Nucleospin Plant II kit (Macherey-Nagel) according to the manufacturer’s instructions. Due to the great variety in mycoflora, PCR amplification concerned the ITS region only; on the other hand, the ITS region is regarded as an efficient barcode for most fungal taxa [37,38,39]. ITS1-ITS4 primers were used for filamentous fungi (including mycelia sterilia too), whereas ITS5-ITS4 primers were used for yeasts and pseudo-yeasts [40]. Further details of the complete identification protocol are reported in Girometta et al. (2020) [41].

### 2.4. Estimation of Ecological Parameters

The wastewater flow in the treatment plants under examination is mainly unidirectional and the two plants share only one significant re-pumping line from Oxy to Denitro.

The most water proceeds from the discharge of the activated sludge to the final depuration stages and discharge in the stream. This allows for the approximation of the data structure to a spatial environmental gradient whose sample selection scheme is directional from a point source [42].

Based on the General concepts of α-, β-, γ- and ζ-diversity, the composition and structure of the communities in each depuration stage were investigated and compared along the depuration process, i.e., stage by stage.

In order to estimate the α-diversity in each depuration stage, Simpson’s evenness and Pielou’s regularity were compared as suggested by Mouillot et Leprêtre (1999) [43] and calculated as summarized by Bullini et al. (1998) [44].

(1) $$E=\sum_{i=1}^{S}p_{i}^{2}/S$$

where: *p_i_* = fraction of individuals of the species *i* in the overall individual population; *S* = overall number of species in the population = γ diversity

As summarized by Baselga (2010) [45], “β-diversity is the variation of species composition of assemblages”, i.e., the variation between depuration stages in this context. The β-diversity partitioning was investigated based on pairwise (nearest neighbor) presence-absence models by Jaccard’s and Simpson’s indices [46].

According to Hui and McGeoch (2014) [47], “ζ-diversity is the number of species shared by a given number of sites and provides a measure of turnover for each combination of *i* sites”. Analogous to β-diversity, ζ-diversity was normalized based on Jaccard’s assumptions [42,45].

As a whole, β-diversity and ζ-diversity were calculated as follows:

Jaccard’s dissimilarity

β_cc_ = (b + c)/(a + b + c) (2)

Jaccard’s distance

β_rich_ = |b − c|/(a + b + c) (3)

Simpson’s turnover

β_−3_ = β_cc_ − β_rich_ (4)

**Figure 2:**
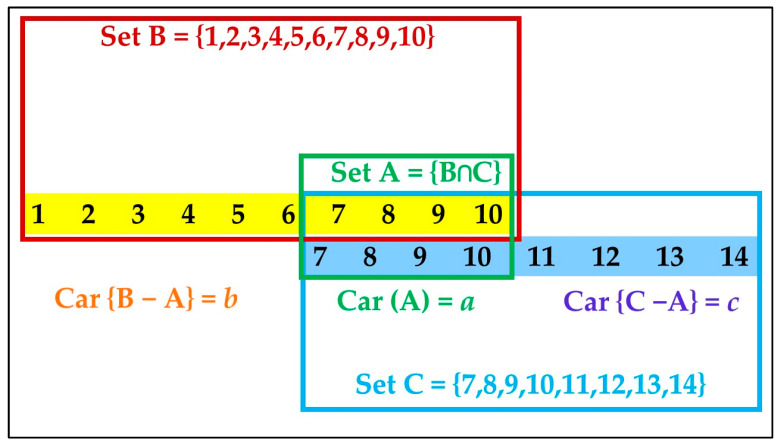
Basic generic example scheme of the data structure as applied in β-diversity and ζ-diversity formulae. The hypothetical example considers two neighbor sites including 10 and eight species, respectively.

Normalized ζ_i_-diversity

(i = 2) = ζ_2_ = a/(a + b + c) (5)

where: a is the number of taxa (cardinality) in set A = {B ∩ C}, i.e., a = Car (A); b = Car {B − A}; and c = Car {C − A}, given two neighbor sites A and B (Figure 2).

## 3. Results and Discussion

### 3.1. Sampling, Isolation in Pure Culture and Identification

From the whole pool of fungal taxa sampled, 60 morphotypes from Plant 1 and 47 from Plant 2 were successfully isolated in pure culture.

The morphotype approach generally fails in discriminating most yeast species from each other, and the same happens for arthrosporigenous pseudo-yeasts. Yeasts and pseudo-yeasts must be therefore sampled more intensely than moulds.

ITS-based molecular identification of the selected strains resulted in acceptable discrimination for all of the morphological categories under examination (yeasts, sporigenous filamentous, mycelia sterilia).

All fungal taxa sampled in this study are reported in Table 3: genera identification was carried out by morphological approach, and species identification was achieved by ITS-based molecular analysis. Taxonomy check on MycoBank [48].

Certain fungal taxa were found in almost every stage of depuration: genera such as *Acremonium*, *Aspergillus*, *Cladosporium*, *Fusarium*, *Mucor*, *Penicillium* and *Trichoderma* are generally found in water and soil and their spores are constantly present in the air [49,50]. These fungi follow the stream across the depuration stages and their availability as environmental contaminants can explain why some taxa are present even after the oxidation and disinfection process, as they are easily sampled by chance.

Family *Trichosporanaceae* is also well represented by species of the genera *Apiotrichum*, *Cutaneotrichosporon* and *Trichosporon*, once all are grouped in the latter [51]. These fungi are yeast and yeast-like organisms generally isolated from soil and environment and some species also from human and animal skin [52]. Species such as *Apiotrichum domesticum*, *Apiotrichum montevideense, Cutaneotrichosporon mucoides* and *Trichosporon asahii* are potentially pathogenic and of clinical importance [52,53] but from this study it emerges that even if these fungi are found in different depuration stages, they are successfully eliminated by the depuration process, as they are no longer found in post-ozonation (1-End) and in Filtration input (2-End).

### 3.2. Diversity Patterns at Fungal Order Scale

**Figure 3:**
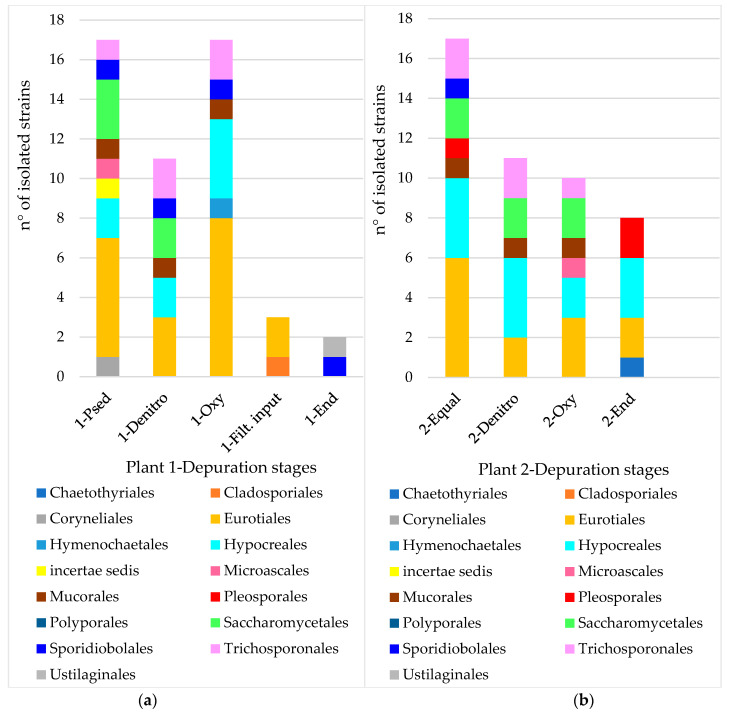
Diversity pattern of the isolated strains at an Order scale in Plant 1 (**a**) and Plant 2 (**b**) with reference to the depuration stage of provenance.

By merging morphotypes with results from molecular identifications, the isolated strains show the diversity pattern reported in Figure 3, giving us the composition of the fungal community throughout both treatment plants. The single data were grouped at the taxonomic level of order to better compare the mycoflora present both in the different depuration stages in the two plants and during the four seasons.

As expected, diversity in wastewater in the first sampling stages (1-PSed and 2-Equalization) is more affected by external propagule sources, both from the urban areas and agricultural systems. Eurotiales, Hypocreales, Saccharomycetales and Trichosporonales are the Orders mainly sampled.

Among all isolated strains, Eurotiales is the most represented in both the treatment plants and in almost all depuration stages. In this study, Eurotiales include *Aspergillus* and *Penicillium or Talaromyces*, as well as *Paecilomyces*. It should be noted that the nomenclatural distinction between *Penicillium* and *Talaromyces* has been adopted, despite the fact that they are the anamorph and teleomorph of the same taxon, respectively. This decision was taken to preserve the information about the occurrence of species which are known to reproduce sexually as well.

Furthermore, Hypocreales constitutes the base of the fungal community in this study, as species belonging to this order have been sampled in three out of five depuration stages in Plant 1 and in all stages in Plant 2. *Fusarium* and *Trichoderma*, which are very common in agricultural systems and soils, are the most represented species. A special mention is deserved by *Trichoderma*, whose species play an important role in soil ecology due to their competition and hyperparasitism versus phytopathogens (mainly fungi and nematodes). Moreover, *Trichoderma* species stimulate plant defense induction [54]. Here, five species were detected: *T. harzianum* and *T. virens*, *T. citrinoviride* and *T. saturnisporum*, and *T. asperellum* (the most common species in the present sampling) [55]. The widespread *T. asperellum* is particularly interesting because only in 1999 it was recognized to be a different species from *T. viride*; however, since then *T. asperellum* has been increasingly detected in agricultural soils. This is likely to also be due to its above mentioned application as a biocontrol agent [56].

Despite the fact that a quantitative approach is out of the scope of the present work, it can be noted that *Fusarium* species are less represented than its major antagonists in the soil, i.e., *Trichoderma* species; this is important since both *F. oxysporum* and *F. fujikuroi* are severe phytopathogens [57].

*Cosmospora*, typically developing an *Acremonium*-like morphology, is phylogenetically close to *Fusarium*. Here, the genus is represented by *C. butyri*, which is related to lipid-rich substrates [58].

According to the recent taxonomic revision of the genus *Paecilomyces*, the species *P. lilacinus* now is named *Purpureocillium lilacinum* and it has switched from Eurotiales to Hypocreales [59].

Saccharomycetales includes fungi that are well-known to be common in wastewater, where they degrade simple polysaccharides and fatty acids. In the present work, eight genera belonging to Saccharomycetales were detected: *Candida*, *Dipodascus*, *Diutina*, *Galactomyces*, *Geotrichum*, *Scheffersomyces*, *Yarrowia* and *Zygoascus*. *Geotrichum*, which is found worldwide in air, soil, water, sewage, as well as in plants, besides being found in human feces, was the most representative, as it concerns morphotype frequency and spatial colonization in a Petri dish, displaying most pseudo-yeast morphology.

The order Trichosporonales resulted in three Genera phylogenetically very close to each other and belonging to *Trichosporon sensu lato*, i.e., *Apiotrichum*, *Cutaneotrichosporon* and *Trichosporon sensu stricto*. *Trichosporon s.l.* morphotype proved to be very common and displayed both budding and arthrospore formation. As for Saccharomycetales, *Trichosporon s.l.* is also commonly represented in soil and in water; however, its trophic spectrum also includes keratinolysis and thus degradation of hair(s) and skin in wastewater [9,60,61].

As a whole, yeasts and pseudo-yeasts generally are over-represented in wastewater treatment plants as they are favored by the abundance of organic matter and, compared to filamentous fungi, are facilitated in growth by the asexual mode of reproduction (buds and arthrospores). Filamentous fungi in continuous wastewater flow are often hampered in sporulation and mycelia can produce forms of resistance such as chlamydospores [62].

### 3.3. Seasonal Variation

**Figure 4:**
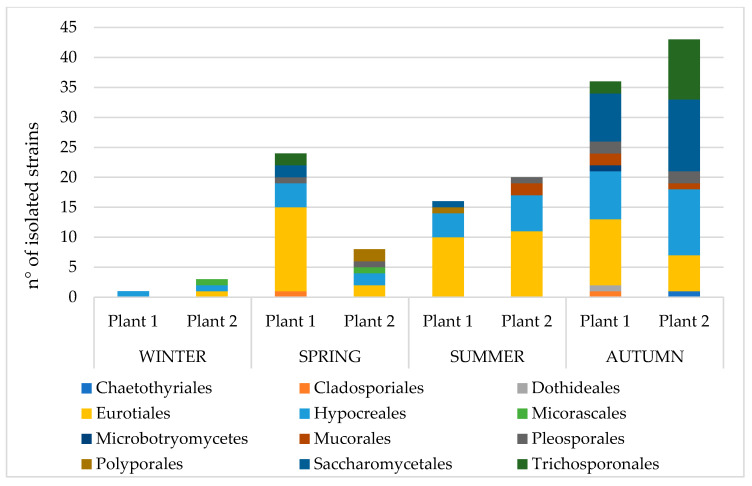
Seasonal variation of the isolated strains at an Order scale.

Fungal community composition at the Order scale shows a seasonal variation, with similar results between the two plants (Figure 4).

A higher number of isolates was found in summer and autumn compared to winter and spring. Orders follow this trend as well.

Eurotiales (mainly *Aspergillus* and *Penicillium* species) and Hypocreales (mainly *Fusarium* and *Trichoderma* species) are confirmed to be the most represented in the two plants across the whole year. Saccharomycetales with *Geotrichum* species and Trichosporanales are also frequent, especially in autumn. Other orders are less represented and were sporadically isolated compared to the others.

As a whole, the wastewater environment seems to host a wider and more diversified community in summer and autumn compared to the other seasons: these results confirm what is as also reported in other works [32,33].

Seasonal variation of isolated strains is probably related to conditions of humidity and temperature, with rainy and warmer months characterized by a more diverse fungal community. This relation is also supported by the metereological data of Lombardia: April, May, June, September, October and November are the months with most average millimeters of precipitation and with average temperatures between 15 °C and 20 °C [63].

### 3.4. Diversity Indices

Since a wastewater treatment plant is composed of different systems and environmental conditions, different community structures are expected in each depuration stage.

Simpson’s evenness and Pielou’s regularity describe how each taxon is representative within the community based on the ratio between the taxon individuals and the overall number of individuals.

**Figure 5:**
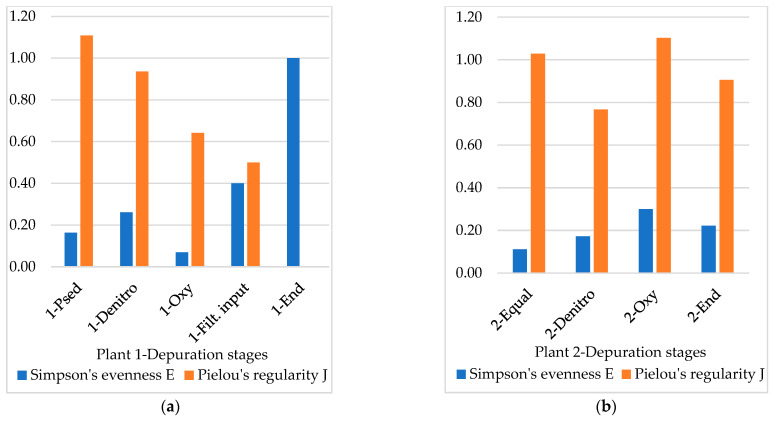
Simpson’s evenness and Pielou’s regularity at an Order scale in different depuration stages of Plant 1 (**a**) and Plant 2 (**b**).

Evenness indices by Simpson (1949) [64] and Pielou (1966) [65] are compared in Figure 5. The substantial disagreement between the two indices suggests that Pielou’s regularity, (a derivation from Shannon-Weaver’s index), is not truly informative in this case since the small sample size highlights the bias [43]. As the community structure evolves towards increasing diversity loss, zero inflation is a bias factor to take into account [66].

Concretely, the different structure of the two plants and sedimentation pools in particular may explain the differences in the taxa occurrence and repartition along the depuration stages. The final stage in Plant 1 loses diversity (Simpson’s evenness 100) with only few species represented, while Plant 2 appears to be favoured in preserving more propagules until the final stages.

**Figure 6:**
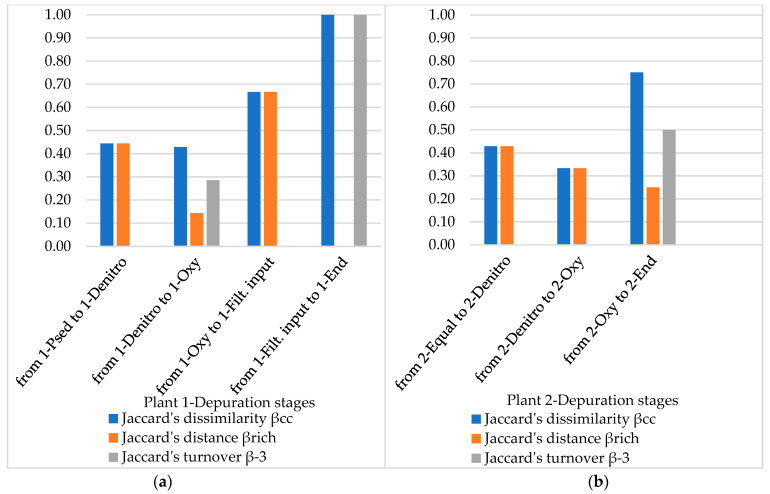
Pairwise distance and directional turnover based on Jaccard’s models at an Order scale in different depuration stages in Plant 1 (**a**) and Plant 2 (**b**).

**Figure 7:**
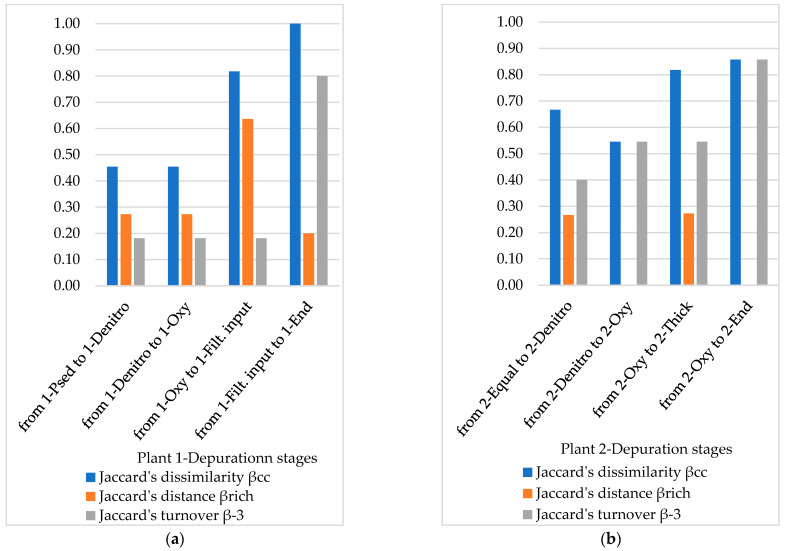
Pairwise distance and directional turnover based on Jaccard’s models at a genus scale in different depuration stages in Plant 1 (**a**) and Plant 2 (**b**).

As mentioned, β-diversity described the compositional change of the community. The above discussed data suggest that β-diversity partitioning is governed by pairwise presence/absence models. Jaccard’s indices and Simpson’s turnover, i.e., normalizations of raw ζ-diversity [42], are reported in Figure 6 and Figure 7 for Order and genus scale, respectively.

Based on the order scale, Jaccard’s dissimilarity β_cc_ increases in Plant 1 by a pairwise comparison of depuration stages, whereas the Jaccard’s turnover β_−3_ based on absolute species number doesn’t show a clear trend, except for the final stage. The dissimilarity trend in Plant 2 is less regular. In Plant 1, the severe constraints before the final discharge provoke a dramatic increasing in turnover. The Jaccard’s distance β_rich_ in Plant 1 is highest when crossing from 1-Oxy to 1-Filt. Input, despite the turnover, is null, as the number of isolated strains is higher compared to the previous stage; however, it is not the same in Plant 2, where the distance is less variable.

As expected, the genus scale is more informative than the order scale, although only four orders are represented by three or more genera. Based on genus scale, Jaccard’s dissimilarity βcc in Plant 1 particularly increases when crossing from 1-Oxy to 1-Filt. input and then furtherly increases to final discharge. More interestingly, Plant 1 has a constantly low Jaccard’s turnover β_−3_ except when crossing to the final discharge, whereas the turnover in Plant 2 is always around 0.5. The Jaccard’s distance β_rich_ is similar among the stages in both the plants, except for the stage from 1-Oxy to to 1-Filt. input in Plant 1 (that explains the most dissimilarity observed by β_cc_) and the stage from 2-Denitro to 2-Oxy in Plant 2 (although the communities are qualitatively different due to turnover). In Plant 2, turnover has a remarkable role in preserving the diversity.

The activated sludge seems therefore to be a critical stage that imposes environmental constraints with particular concern to oxygenation. In many depuration plants there are two backward re-pumping lines: the first is from the oxidation pool to the denitrification one (this is both the case of Plant 1 and Plant 2), and the second is from the final filtration to the denitrification one (this is the case of Plant 1 only). Such a partial bi-directional flow favors the community homogenization at least between the activated sludge and the sedimentation stage.

Notwithstanding this, the stage from the activated sludge to the secondary sedimentation provokes, as mentioned, a dramatic decline in microbial richness, since the supernatant is impoverished in nutrients with respect to the sunk slurry particles. As expected, and most important in the depuration process, the final discharge furtherly destroys the microbial community in the water. As a whole, such a loss can be seen both as the result of microbial quantitative reduction and consequent qualitative sampling bias.

This is consistent with normalized ζ-diversity at a genus scale that clearly shows similar dynamics in Plant 1 and Plant 2. The stage from the initial input to the activated sludge represents a first bottleneck more in Plant 2 than in the Plant 1. The ζ-diversity is in fact lower when passing from the 2-Equal to 2-Denitro (ζ-diversity 0.33) than from 1-PSed to 1-Denitro (ζ-diversity 0.55). This was unexpected because the wastewater in 2-Equal is very similar to 2-Denitro, whereas 1-Psed is a further intermediate stage between the initial input and the denitrification.

In both the treatment plants the output from the activated sludge (1-Oxy and 2-Oxy) encounters a bottleneck where the number of shared taxa (i.e., ζ-diversity) crashes (Plant 1 ζ-diversity 0.18; Plant 2 ζ-diversity 0.14). In Plant 1 the output from the activated sludge undergoes secondary sedimentation; the resulting supernatant (1-Filt. input) is therefore significantly depleted of particles as well as fungal propagules. As a whole, ζ-diversity between 1-Oxy and 1-Filt. input relies on sharing taxa in Eurotiales (Figure 3).

In Plant 2, the output from the activated sludge undergoes two different processes which result in similar values of ζ-diversity. When passing from 2-Oxy to 2-End ζ-diversity relies on sharing taxa in Hypocreales and Eurotiales (i.e., true moulds), which are very common in the environment and may therefore be represented even at the exit of the depuration process.

Actually, the taxa sampled after the ozonation process are to be meant as sampled by chance due to the environmental availability of propagules outside the ozonation compound itself instead of the failure of the depuration process. It should be kept in mind that ζ-diversity is a similarity measure based on diversity instead of population size, therefore it does not at all imply considerations about the depuration success.

The depuration principle as meant by Italian law [2,6] aims at “disinfection” instead of “sterilization”, meaning that microbial contamination is accepted on condition it is below the safety threshold indicated by the law itself. Nevertheless, it is noteworthy that such thresholds concern bacteria only (namely the coliforms *Escherichia coli*, *Enterococcus* spp., *Clostridium perfringens*, and *Pseudomonas aeruginosa*) whereas no fungal propagules are monitored by default [67]. This is due to the fact that the depuration process basically relies on the bacterial activity more than the fungal one. Moreover, there is another issue hampering the ability to apply to the fungi the same qualitative and quantitative surveys routinely applied to bacteria: filamentous fungi grow much slower even in optimal conditions [68]. However, as previously discussed, filamentous fungi show a severely limited reproduction in sludges and slurries of depuration plants, whereas yeasts and pseudo-yeasts are more favoured but their populations are crashed by the conventional disinfection methods [6] as well as the bacterial ones, particularly when adopting sieving biomembranes [34,69]. This means that the discharged water from a depuration plant provides a negligible fungal inoculum into the receiving stream. Nevertheless, periodic surveys on the fungal propagules in the discharged water may suggest what is the most efficient disinfection method when projecting future plants or restructuring/adjusting the existent ones.

## 4. Conclusions

Wastewater treatment plants are composite systems where different fungal communities are hosted depending on the specific conditions of each depuration stage. The diversity pattern in input strongly affects the community in first stages (primary sedimentation and activated sludge), but is radically changed in secondary sedimentation, i.e., after the activated sludge stage and the separation of spent microbial particles and residual nutrients from the supernatant. As expected, the cyclic flow between denitrification and nitrification systems contributes to homogenize the communities in activated sludge despite the difference in oxidation conditions.

From a qualitative-mycofloristic perspective, Eurotiales, Hypocreales and Trichosporonales, as well as Saccharomycetales, are the most represented orders in all the depuration stages, mainly including genera such as *Penicillium* or *Talaromyces*, *Aspergillus*, *Trichoderma*, *Trichosporon sensu lato* and several yeasts and pseudo-yeasts such as *Geotrichum*.

Despite the fact that Plant 1 and Plant 2 show different diversity patterns, the above mentioned taxa are basically represented in both.

The ITS region approach resulted in acceptable discrimination based on cross-check on the output by Mycobank Molecular ID. ITS is regarded as a suitable barcode region when dealing with surveys on a wide spectrum of fungi. Further selected markers may be introduced to confirm specific identification within complex Genera such as *Penicillium* or *Talaromyces* and *Trichoderma* as well as to investigate sub-specific diversity.

The wastewater fungal community is an often ignored, but equally represented, part of the microbial community. Deepening the knowledge about fungal species’ presence and fluctuation across depuration stages and seasons can help in better understanding their role in the depuration process and how to exploit them in synergy with the bacterial component. This work also highlights the importance of periodic sampling campaigns to monitor the fungal community not only in the different depuration stages but also into the final water stream.

## Figures and Tables

**Table 1:** Treatment stages examined in this study.

Scheme	Plant	Code in This Study
Primary sedimentation of input wastewater	1	1-PSed
Activated sludge–denitrification	1	1-Denitro
Activated sludge–oxidation	1	1-Oxy
Filtration input–discharge post-secondary sedimentation	1	1-Filt. input
Post ozonation	1	1-End
Equalization of input wastewater	2	2-Equal
Activated sludge–denitrification	2	2-Denitro
Activated sludge–oxidation	2	2-Oxy
Filtration input	2	2-End

**Table 2:** Dilution scheme for wastewater and sludge samples.

Stage	Bulk	1:10	1:100	1:1000	1:10,000
1-PSed	x	x	x	x	
1-Denitro			x	x	
1-Oxy		x	x	x	
1-Filt. input			x	x	x
1-End			x	x	x
2-Equal	x	x	x	x	
2-Denitro			x	x	
2-Oxy		x	x	x	
2-End			x	x	x

**Table 3:** Sampled fungal taxa with reference to the depuration stage of provenance.

Fungal Taxa	Author	Depuration Stages of Provenance
*Acremonium* spp.	Link	1-Psed, 1-Denitro, 1-End; 2-Equal, 2-Denitro, 2-Oxy
*Alternaria* spp.	Nees	1-Denitro, 2- Equal
*Apiotrichum domesticum*	(Sugita, A. Nishikawa & Shinoda) Yurkov & Boekhout	2-Oxy
*Apiotrichum montevideense*	(L.A. Queiroz) Yurkov & Boekhout	2-Equal
*Apiotrichum laibachii*	(Windisch) Yurkov & Boekhout	1-Psed, 1-Denitro, 1-Oxy
*Aspergillus flavus*	Link	1-Psed, 1-Denitro, 1-Oxy, 1-Filt. Input; 2-Equal, 2-Denitro, 2-Oxy
*Aspergillus fumigatus*	Fresen.	1-Psed, 1-Denitro, 1-Oxy, 1-Filt. Input, 1-End; 2-Equal, 2-Denitro, 2-Oxy, 2-End
*Aspergillus niger*	Tiegh.	1-Psed, 1-Oxy, 1-Filt. Input, 1-End; 2-Equal, 2-Denitro, 2-Oxy
*Aspergillus tubingensis*	Mosseray	1-Oxy
*Aspergillus* spp.	P. Micheli ex Haller	1-Psed, 1-Denitro, 1-Oxy, 1-Filt. Input; 2-Oxy, 2-End
*Candida pseudolambica*	M.T. Sm. & Poot	1-Denitro
*Cladosporium* spp.	Link	1-Psed, 1-Denitro, 1-Oxy, 1-Filt. Input, 1-End; 2-Equal, 2-Denitro, 2-Oxy, 2-End
*Chaetomium* sp.	Kunze	2-Oxy
*Chrysosporium tropicum*	J.W. Carmich.	1-Denitro, 1-Oxy, 1-Filt. Input
*Cosmospora butyri*	(J.F.H. Beyma) Gräfenhan	2-Equal
*Cutaneotrichosporon cutaneum*	(Beurm., Gougerot & Vaucher bis) Xin Zhan Liu, F.Y. Bai, M. Groenew. & Boekhout	1-Psed, 1-Denitro, 1-Oxy, 1-Filt. Input; 2-Equal, 2-Oxy
*Cutaneotrichosporon jirovecii*	(Frágner) Xin Zhan Liu, F.Y. Bai	1-Oxy; 2-Equal, 2-Oxy
*Cutaneotrichosporon mucoides*	(E. Guého & M.T. Sm.) Xin Zhan Liu, F.Y. Bai, M. Groenew. & Boekhout	1-Psed, 1-Denitro, 1-Oxy
*Debaryomyces hansenii*	(Zopf) Lodder & Kreger	1-Psed
*Dipodascus fermentans*	(Diddens & Lodder) P.M. Kirk	1-Denitro
*Diutina neorugosa*	(Paredes, Deanna A. Sutton, Cano & Guarro) Khunnamw., Jindam., Limtong & Lachance	1-Psed
*Engyodontium* sp.	de Hoog	2-Equal
*Exophiala lecanii-corni*	(Benedek & G. Specht) Haase & de Hoog	2-Equal, 2-End
*Fusarium fujikuroi*	Nirenberg	1-Oxy
*Fusarium oxysporum*	Schltdl.	2-Denitro
*Fusarium* spp.	Link	1-Psed, 1-Denitro, 1-Oxy, 1-Filt. Input; 2-Equal, 2-Denitro, 2-Oxy, 2-End
*Fusicladium* sp.	Bonord.	2-Equal
*Geotrichum candidum*	Link	1-Psed, 1-Denitro, 1-Oxy, 1-Filt. Input, 1-End; 2-Equal, 2-Denitro, 2-Oxy, 2-End
*Geotrichum fragrans*	Morenz	2-Oxy
*Geotrichum* spp.	Link	1-Psed, 1-Denitro, 1-Oxy 2-Equal, 2-Denitro, 2-Oxy, 2-End
*Graphium* sp.	Corda	1-Psed
*Mucor* spp.	Fresen.	1-Psed, 1-Denitro, 1-Oxy; 2-Equal, 2-Denitro, 2-Oxy, 2-End
*Oxyporus latemarginatus*	(Durieu & Mont.) Donk	1-Oxy
*Phialophora* spp.	Medlar	1-Psed, 1-Denitro, 1-Oxy, 1-Filt. Input
*Phycomyces* sp.	Kunze	1-Psed, 1-Denitro, 1-Oxy, 1-Filt. Input
*Phoma* spp.	Sacc.	1-Denitro, 1-Filt. Input; 2-Equal
*Penicillium albocoremium*	(Frisvad) Frisvad	1-Oxy
*Penicillium crustosum*	Thom	2-Equal
*Penicillium expansum*	Link	2-Oxy
*Penicillium griseofulvum*	Dierckx	1-Oxy, 1-Equal
*Penicillium olsonii*	Bainier & Sartory	1-Psed
*Penicillium verrucosum*	Dierckx	1-Oxy
*Penicillium* spp.	Link	1-Psed, 1-Denitro, 1-Oxy, 1-Filt. Input, 1-End; 2-Equal, 2-Denitro, 2-Oxy
*Purpureocillium lilacinum*	(Thom) Luangsa-ard, Houbraken, Hywel-Jones & Samson	1-Oxy; 2- Equal, 2-Denitro, 2-Oxy, 2-End
*Rhizopus oryzae*	Went & Prins. Geerl.	1-Psed, 1-Denitro, 1-Oxy; 2-Equal, 2-Denitro, 2-Oxy
*Rhodotorula glutinis*	(Fresen.) F.C. Harrison	1-Psed, 1-Denitro, 1-Oxy, 1-End; 2-Equal, 2-End
*Rhodotorula mucilaginosa*	(A. Jörg.) F.C. Harrison	1-Oxy
*Sampaiozyma ingeniosa*	(Di Menna) Q.M. Wang, F.Y. Bai, M. Groenew. & Boekhout	1-Psed
*Scedosporium dehoogi*	Gilgado, Cano, Gené & Guarro	2-Oxy
*Scheffersomyces spartinae*	(Ahearn, Yarrow & Meyers) Kurtzman & M. Suzuki	1-Denitro
*Scopulariopsis brevicaulis*	(Sacc.) Bainier	1-Psed, 1-Denitro; 2-Equal, 2-Denitro, 2-Oxy, 2-End
*Sporobolomyces* spp.	Kluyver & C.B. Niel	2-Equal 2-Denitro
*Talaromyces flavus*	(Klöcker) Stolk & Samson	2-Equal
*Talaromyces* spp.	C.R. Benj.	2-Equal
*Trichoderma asperellum*	Samuels, Lieckf. & Nirenberg	1-Psed, 1-Denitro, 1-Oxy; 2-Equal, 2-Oxy
*Trichoderma citrinoviride*	Bissett	2-Oxy
*Trichoderma harzianum*	Rifai	1-Psed
*Trichoderma saturnisporum*	Hammill	2-Denitro
*Trichoderma virens*	(J.H. Mill., Giddens & A.A. Foster) Arx	1-Denitro
*Trichoderma* spp.	Pers.	1-Psed, 1-Denitro, 1-Oxy, 1-End; 2-Equal, 2-Denitro, 2-Oxy, 2-End
*Trichosporon asahii*	Akagi ex Sugita, A. Nishikawa & Shinoda	1-Psed, 1-Denitro, 1-Oxy, 1-Filt. Input
*Verticillium* sp.	Nees	2-Oxy
*Yarrowia lipolytica*	(Wick., Kurtzman & Herman) Van der Walt & Arx	2-Denitro
*Zygoascus polysorbophila*	(Kurtzman) Nagats., Kiyuna & Sugiy.	2-Denitro
Other yeasts		1-Psed, 1-Denitro, 1-Oxy, 1-Filt. Input, 1-End; 2-Equal, 2-Denitro, 2-End
Sporigenous fungi		1-Psed, 1-Denitro, 1-Oxy, 1-Filt. Input
Mycelia sterilia		1-Psed, 1-Oxy, 1-Filt. Input; 2-Denitro

## Data Availability

The data presented in this study are available on request from the corresponding author. The data are not publicly available due to industrial secrecy.

## References

[1] Alleman J.E., Prakasam T.B.S. (1983). Reflections on seven decades of activated sludge history. J. Water Pollut. Control. Fed..

[2] Decreto Legislativo 3 Aprile 2006, n. 152 Norme in Materia Ambientale. Gazzetta Ufficiale della Repubblica Italiana n.88 del 14-4-2006—Suppl. Ordinario n. 96. https://www.gazzettaufficiale.it/dettaglio/codici/materiaAmbientale.

[3] Istituto Superiore per la Protezione e la Ricerca Ambientale. https://www.isprambiente.gov.it/.

[4] Behnamia A., Benisb K.Z., Shakerkhatibic M., Derafshid S., Sabere A.B., Akbarif N.A.R., Yousefia R. (2018). Comparative study on fungal communities of full scale municipal and industrial wastewater treatment plants. Desalination Water Treat..

[5] Niu L., Li Y., Xu L., Wang P., Zhang W., Wang C., Cai W., Wang L. (2017). Ignored fungal community in activated sludge wastewater treatment plants: Diversity and altitudinal characteristics. Environ. Sci. Pollut. Res..

[6] ANPA—Dipartimento Prevenzione e Risanamento Ambientali (2001). Guida alla Progettazione dei Sistemi di Collettamento e Depurazione delle Acque Reflue Urbane.

[7] Molina-Muñoz M., Poyatos J., Sánchez-Peinado M., Hontoria E., González-López J., Rodelas B. (2009). Microbial community structure and dynamics in a pilot-scale submerged membrane bioreactor aerobically treating domestic wastewater under real operation conditions. Sci. Total Environ..

[8] Wan C.-Y., De Wever H., Diels L., Thoeye C., Liang J.-B., Huang L.-N. (2011). Biodiversity and population dynamics of microorganisms in a full-scale membrane bioreactor for municipal wastewater treatment. Water Res..

[9] Matsunaga K., Kubota K., Harada H. (2014). Molecular Diversity of Eukaryotes in Municipal Wastewater Treatment Processes as Revealed by 18S rRNA Gene Analysis. Microbes Environ..

[10] Yang Y., Wang L., Xiang F., Zhao L., Qiao Z. (2020). Activated Sludge Microbial Community and Treatment Performance of Wastewater Treatment Plants in Industrial and Municipal Zones. Int. J. Environ. Res. Public Health.

[11] Urban Waste Water Treatment Directive (1991). Directive 91/271/EEC of 21 May 1991 concerning urban waste-water treatment. J. Eur. Commun..

[12] Philippot L., Hallin S. (2005). Finding the missing link between diversity and activity using denitrifying bacteria as a model functional community. Curr. Opin. Microbiol..

[13] Ji B., Yang K., Zhu L., Jiang Y., Wang H., Zhou J., Zhang H. (2015). Aerobic denitrification: A review of important advances of the last 30 years. Biotechnol. Bioprocess Eng..

[14] Mohsenpour S.F., Hennige S., Willoughby N., Adeloye A., Gutierrez T. (2021). Integrating micro-algae into wastewater treatment: A review. Sci. Total Environ..

[15] Hyde K.D., Xu J., Rapior S., Jeewon R., Lumyong S., Niego A.G.T., Abeywickrama P.D., Aluthmuhandiram J.V.S., Brahamanage R.S., Brooks S. (2019). The amazing potential of fungi: 50 ways we can exploit fungi industrially. Fungal Divers..

[16] Selbmann L., Egidi E., Isola D., Onofri S., Zucconi L., de Hoog G.S., Chinaglia S., Testa L., Tosi S., Balestrazzi A. (2013). Biodiversity, evolution and adaptation of fungi in extreme environments. Plant Biosyst. Int. J. Deal. All Asp. Plant Biol..

[17] Hillmann F., Shekhova E., Kniemeyer O. (2015). Insights into the cellular responses to hypoxia in filamentous fungi. Curr. Genet..

[18] Harms H., Schlosser D., Wick L.Y. (2011). Untapped potential: Exploiting fungi in bioremediation of hazardous chemicals. Nat. Rev. Microbiol..

[19] Asif M.B., Hai F.I., Singh L., Price W.E., Nghiem L.D. (2017). Degradation of Pharmaceuticals and Personal Care Products by White-Rot Fungi—A Critical Review. Curr. Pollut. Rep..

[20] Zahmatkesh M., Spanjers H., van Lier J.B. (2018). A novel approach for application of white rot fungi in wastewater treatment under non-sterile conditions: Immobilization of fungi on sorghum. Environ. Technol..

[21] Sánchez C. (2020). Fungal potential for the degradation of petroleum-based polymers: An overview of macro- and microplastics biodegradation. Biotechnol. Adv..

[22] Singh R.K., Tripathi R., Ranjan A., Srivastava A.K. (2019). Fungi as potential candidates for bioremediation. Abatement of Environmental Pollutants.

[23] Benguenab A., Chibani A. (2021). Biodegradation of petroleum hydrocarbons by filamentous fungi (Aspergillus ustus and Purpureocillium lilacinum) isolated from used engine oil contaminated soil. Acta Ecol. Sin..

[24] Gadd G.M. (1994). Interactions of Fungi with Toxic Metals.

[25] Abbas S.H., Ismail I.M., Mostafa T.M., Sulaymon A.H. (2014). Biosorption of heavy metals: A review. J. Chem. Sci. Technol..

[26] Cecchi G., Marescotti P., Di Piazza S., Zotti M. (2017). Native fungi as metal remediators: Silver myco-accumulation from metal contaminated waste-rock dumps (Libiola Mine, Italy). J. Environ. Sci. Health Part B.

[27] Cecchi G., Vagge G., Cutroneo L., Greco G., Di Piazza S., Faga M., Zotti M., Capello M. (2019). Fungi as potential tool for polluted port sediment remediation. Environ. Sci. Pollut. Res..

[28] More T., Yan S., Tyagi R., Surampalli R. (2010). Potential use of filamentous fungi for wastewater sludge treatment. Bioresour. Technol..

[29] Kacprzak M., Neczaj E., Okoniewska E. (2005). The comparative mycological analysis of wastewater and sewage sludges from selected wastewater treatment plants. Desalination.

[30] Awad M.F., Kraume M. (2011). Fungal diversity in activated sludge from membrane bioreactors in Berlin. Can. J. Microbiol..

[31] Zhang H., Feng J., Chen S., Li B., Sekar R., Zhao Z., Jia J., Wang Y., Kang P. (2018). Disentangling the Drivers of Diversity and Distribution of Fungal Community Composition in Wastewater Treatment Plants Across Spatial Scales. Front. Microbiol..

[32] Wei Z., Liu Y., Feng K., Li S., Wang S., Jin D., Zhang Y., Chen H., Yin H., Xu M. (2018). The divergence between fungal and bacterial communities in seasonal and spatial variations of wastewater treatment plants. Sci. Total Environ..

[33] Zhang S., Fan F., Meng F. (2020). Seasonality and Community Separation of Fungi in a Municipal Wastewater Treatment Plant. Appl. Environ. Microbiol..

[34] Istituto Regionale di Ricerca della Lombardia (IRER) (2010). Depurazione delle Acque Reflue Urbane: Tecnologie Innovative Idonee Acontesti Molto Urbanizzati. Rapporto Finale.

[35] Lacap D.C., Hyde K.D., Liew E.C.Y. (2003). An evaluation of the fungal ‘morphotype’ concept based on ribosomal DNA sequences. Fungal Divers..

[36] Sutherland W.J. (2006). Ecological Census Techniques: A Handbook.

[37] Nilsson R.H., Ryberg M., Abarenkov K., Sjã¶kvist E., Kristiansson E. (2009). The ITS region as a target for characterization of fungal communities using emerging sequencing technologies. FEMS Microbiol. Lett..

[38] Schoch C.L., Seifert K.A., Huhndorf S., Robert V., Spouge J.L., Levesque C.A., Chen W., Bolchacova E., Fungal Barcoding Consortium, Fungal Barcoding Consortium Author List (2012). Nuclear ribosomal internal transcribed spacer (ITS) region as a universal DNA barcode marker for Fungi. Proc. Natl. Acad. Sci. USA.

[39] Lücking R., Aime M.C., Robbertse B., Miller A.N., Ariyawansa H.A., Aoki T., Cardinali G., Crous P.W., Druzhinina I.S., Geiser D.M. (2020). Unambiguous identification of fungi: Where do we stand and how accurate and precise is fungal DNA barcoding?. IMA Fungus.

[40] White T.J., Bruns T., Lee S., Taylor J., Innis M.A., Gelfand D.H., Sninsky J.J., White T.J. (1990). Amplification and direct sequencing of fungal ribosomal RNA genes for phylogenetics. PCR Protocols: A Guide to Methods and Applications.

[41] Girometta C.E., Bernicchia A., Baiguera R.M., Bracco F., Buratti S., Cartabia M., Picco A.M., Savino E. (2020). An Italian Research Culture Collection of Wood Decay Fungi. Diversity.

[42] McGeoch M.A., Latombe G., Andrew N.R., Nakagawa S., Nipperess D.A., Roigé M., Marzinelli E.M., Campbell A.H., Vergés A., Thomas T. (2019). Measuring continuous compositional change using decline and decay in zeta diversity. Ecology.

[43] Mouillot D., Lepretre A. (1999). A comparison of species diversity estimators. Res. Popul. Ecol..

[44] Bullini L., Pignatti S., De Santo A.V. (1998). Ecologia Generale.

[45] Baselga A. (2010). Partitioning the turnover and nestedness components of beta diversity. Glob. Ecol. Biogeogr..

[46] Baselga A., Leprieur F. (2015). Comparing methods to separate components of beta diversity. Methods Ecol. Evol..

[47] Hui C., McGeoch M.A. (2014). Zeta Diversity as a Concept and Metric That Unifies Incidence-Based Biodiversity Patterns. Am. Nat..

[48] Mycobank. www.mycobank.org.

[49] Domsch K.H., Gams W., Anderson T.H. (1980). Compendium of Soil Fungi.

[50] Ziaee A., Zia M., Goli M. (2018). Identification of saprophytic and allergenic fungi in indoor and outdoor environments. Environ. Monit. Assess..

[51] Liu X.Z., Wang G.M., Göker M., Groenewald M., Kachalkin A.V., Lumbsch H.T., Millanes A.M., Wedin M., Yurkov A.M., Bai F.Y. (2015). Towards an integrated phylogenetic classification of the Tremellomycetes. Stud. Mycol..

[52] de Hoog G.S., Guarro J., Gené J., Figueras M.J. (2005). Atlas of Clinical Fungi.

[53] Montoya Mendoza A.M., González González G.M. (2014). Trichosporon spp.: An emerging fungal pathogen. Med. Univ..

[54] Singh A., Shukla N., Kabadwal B., Tewari A., Kumar J. (2018). Review on Plant-Trichoderma-Pathogen Interaction. Int. J. Curr. Microbiol. Appl. Sci..

[55] Kubicek C.P., Steindorff A.S., Chenthamara K., Manganiello G., Henrissat B., Zhang J., Cai F., Kopchinskiy A.G., Kubicek E.M., Kuo A. (2019). Evolution and comparative genomics of the most common Trichoderma species. BMC Genom..

[56] Gerin D., Pollastro S., Raguseo C., De Miccolis Angelini R.M., Faretra F. (2018). A ready-to-use single-and duplex-TaqMan-qPCR assay to detect and quantify the biocontrol agents *Trichoderma asperellum* and *Trichoderma gamsii*. Front. Microbiol..

[57] Win T.T., Bo B., Malec P., Khan S., Fu P. (2021). Newly isolated strain of Trichoderma asperellum from disease suppressive soil is a potential bio-control agent to suppress Fusarium soil borne fungal phytopathogens. J. Plant Pathol..

[58] Gräfenhan T., Schroers H.-J., Nirenberg H., Seifert K. (2011). An overview of the taxonomy, phylogeny, and typification of nectriaceous fungi in Cosmospora, Acremonium, Fusarium, Stilbella, and Volutella. Stud. Mycol..

[59] Seifert K.A., Gams W. (2011). The Genera of Hyphomycetes—2011 update. Pers. Mol. Phylogeny Evol. Fungi.

[60] Dragičević T., Hren M., Gmajnić M., Pelko S., Kungulovski D., Kungulovski I., Čvek D., Frece J., Markov K., Delaš F. (2010). Biodegradation of Olive Mill Wastewater by Trichosporon Cutaneum and Geotrichum Candidum. Arch. Ind. Hyg. Toxicol..

[61] Kurtzman C., Fell J.W., Boekhout T. (2011). The Yeasts: A Taxonomic Study.

[62] Samuels G.J., Hebbar P.K. (2015). Developing Trichoderma-based products for application in agriculture. Trichoderma Identification and Agricultural Applications.

[63] ARPA-Lombardia. www.arpalombardia.it.

[64] Simpson E.H. (1949). Measurement of diversity. Nature.

[65] Pielou E.C. (1966). The measurement of diversity in different types of biological collections. J. Theor. Biol..

[66] Hasan M.T., Sneddon G., Ma R. (2017). Modeling binomial amphibian roadkill data in distance sampling while accounting for zero-inflation, serial correlation and varying cluster sizes simultaneously. Environ. Ecol. Stat..

[67] Bonadonna L., Musmeci L. (2014). Metodi Analitici di Riferimento per la Valutazione Microbiologica dei Fanghi di Depurazione e di Matrici ad Essi Assimilabili.

[68] Rousk J., Bååth E. (2011). Growth of saprotrophic fungi and bacteria in soil. FEMS Microbiol. Ecol..

[69] Konieczny K. (2015). Effectiveness of wastewater treatment with the use of the biological membrane reactors. Annu. Set—Environ. Prot..

